# Dietary and Exercise Interventions for Perimenopausal Women: A Health Status Impact Study

**DOI:** 10.3389/fnut.2021.752500

**Published:** 2022-01-27

**Authors:** Shuping Hao, Sisi Tan, Jing Li, Weimin Li, Jingyun Li, Xiaochen Cai, Zhongxin Hong

**Affiliations:** ^1^Department of Clinical Nutrition, Pinggu Hospital, Beijing Friendship Hospital Affiliated to Capital Medical University, Beijing, China; ^2^Department of Clinical Nutrition, Beijing Friendship Hospital, Capital Medical University, Beijing, China

**Keywords:** body composition, dietary interventions, perimenopausal period, resistance motion, health education

## Abstract

**Purpose:**

To explore the impact of different intervention methods on physical health indexes of perimenopausal women.

**Methods:**

Seventy-eight perimenopausal women were divided into three groups. Group A received routine gynecological diagnosis and treatment and participated in centralized lifestyle health education. Group B was required to participate in all intensive education content, and professional dietitians gave individualized dietary guidance. Group C received intensive education, individualized diet intervention and intensified resistance exercise. Dietary scores, exercise habits, waist circumference, body mass index, fat and muscle mass were observed after three months.

**Results:**

After the intervention, the total diet score of group C was higher than groups A and B, and the red meat score was significantly reduced. The number of patients in groups B and C who exercised >3 times per week and the number of resistance exercises were significantly increased, while the number of aerobic exercises per week in group C was significantly increased. The body fat and waist circumference were significantly reduced, and skeletal muscle weight was significantly increased. Body mass index, trunk fat ratio and waist-to-hip ratio were significantly decreased in groups B and C, while trunk muscle was significantly increased in group C.

**Conclusion:**

The combined intervention of the three methods can give full play to the synergistic effect of various interventions. The improvement effect of increasing the appropriate amount of resistance exercise is more obvious, which is worthy of further promotion and application.

## Introduction

The perimenopausal period refers to the first signs of ovarian function decline until one year after the last menstruation in women. It refers to the transitional period from maturity to old age in women. The clinical manifestations of menstrual disorders are menopause, memory loss, irritability, tidal sweating, insomnia, joint pain and so on (1). Estrogen deficiency in postmenopausal women will damage the normal bone turnover cycle, thus increasing the prevalence of osteoporosis and fractures (2). In addition, estrogen regulates fat distribution and adipocyte differentiation and increases visceral fat, leading to increased risk of chronic diseases such as coronary heart disease and diabetes (3). Therefore, the proper conditioning of perimenopausal women's bodies is significant in improving the symptoms and preventing chronic diseases. Zhu's study found that diet and exercise therapy for obese postmenopausal women can significantly improve blood lipid levels, improve insulin resistance, reduce plasma insulin levels and effectively prevent type 2 diabetes (4). Zhang et al. reviewed the therapeutic effect of exercise rehabilitation on perimenopausal syndrome (5). Moreover, the standard diet of perimenopausal women should pay attention to energy balance, sufficient calcium and vitamin D, sufficient dietary fiber and an appropriate amount of iron (6). Therefore, the purpose of this study is to explore the dietary and/or exercise intervention for perimenopausal women, explore the impact of different intervention methods on their physical health indicators and provide a valuable reference for their physical conditioning.

## Materials and Methods

### Inclusion and Exclusion Criteria

Inclusion criteria: ① females with clear perimenopausal diagnoses; ② no systematic nutrition education; able to walk without assistance; ③ able to communicate normally. Exclusion criteria: ① having taken drugs for weight loss treatment within three months; ② having suffered from myocardial infarction, unstable cardiovascular disease or other diseases in the past three months; ③ chronic renal insufficiency or renal failure; ④ patients with diabetes on insulin treatment; ⑤ patients with upper or lower limb fractures or other exercise contraindications in the past three months; ⑥ neuromuscular diseases or taking drugs that affect neuromuscular function; ⑦ taking drugs that include hormone replacement medication or other drugs that could affect body composition.

### Research Subjects

Seventy-eight perimenopausal women seen at the gynecology outpatient department of Beijing Pinggu Hospital from June 2018 to August 2018 were recruited as the research subjects, with an average age of 48.65 ± 3.06 years old. They were randomly divided into three groups, with 32 cases in each group. After the implementation of the study, some participants were unwilling to participate in the follow-up observation after attending intensive education. Group A was the collective education group with 18 cases and 14 dropped off. Group B was the individualized diet group with 28 cases, two suffered sudden illness and two had temporary withdrawal. Group C was the individualized diet + resistance exercise group with 32 cases. Seventy-eight patients completed all observation items, and the shedding rate was 18.75%. The Ethics Committee of our hospital reviewed this study, and all participants signed informed consent.

### Methods of Intervention

#### Group Intervention

Group A received routine gynecological diagnoses and treatments and participated in lectures by nutritionists, pharmacists and nurses. They learned about the characteristics of menopausal nutrition and metabolism, the meaning and principles of diet intervention, exercise methods and precautions, drug selection and administration methods. In addition to collective education, Group B followed individualized dietary guidance according to the DASH diet principles, recorded a diet diary three days a week and uploaded it to WeChat in time. Any unreasonable diet structure was corrected and demonstration meals were provided once a month. Group C received collective education and individualized dietary interventions. At the same time, professional sports coaches conducted on-site training and guidance. After three months of intervention, the changes in various observation indicators were reviewed.

#### Diet Score

This study was improved based on the DASH diet score method of Shao et al. (7) and established a diet score based on the classification of 13 main foods. The main guiding principles of the 13 foods were sufficient fruits, vegetables, low-fat dairy products, moderate amounts of nuts and beans, white meats such as poultry, fish and shrimp, whole grains and potatoes, reduced fat and animal organs, controlling salt intake, red and processed meats, desserts and sweet drinks. For fruits, vegetables, nuts and beans, low-fat dairy products and whole grains, the lowest intake frequency was given 0 points, the highest was 4 points, and the middle was 1 to 3 points. For sweet drinks, red, processed and fatty meats and animal offal, the lowest intake frequency and salt intake are given 4 points; the highest intake frequency and salt intake are 0 points, and the middle is 1 to 3 points. Finally, the total scores of the 13 food categories were added to obtain the diet score.

### The Exercise Mode Was Guided by the Sports Coach On-Site

Aerobic and resistance exercise can improve cardiovascular function and heart rate variability in perimenopausal women (8). Every Monday and Thursday, it concentrated on strengthening resistance exercises (6), 30–40 min each time. The exercise process design: prepare for five minutes → upper limb dumbbell weight series exercises for 15 min → lower limb squats, squat walking for 15 min → stretching and finishing for five minutes; aerobic exercise more than two days a week, brisk walking or jogging 8,000–10,000 steps a day, and record exercise frequency.

### Observation Indicators

#### General Information

It includes name, age, occupation, education, education level, waist circumference, height, production status, menopause status, exercise status, past medical history, including hypertension, diabetes and hyperlipidaemia, liver and kidney disease, etc., and medication status.

For height (m), a height range finder was used (Jiangsu Suhong Medical Instruments Co., Ltd., license number, Su made 00000600), shoes were removed, and after strict calibration, the result was accurate to 0.1 cm.

Waist circumference (cm) was measured at the midpoint between the ribs and the iliac ridge using a soft ruler made of non-stretchable material, and the scale was read to 0.1 cm.

Body composition measurements used the InBody 770 Body Composition Analyser (South Korea's Basbes Medical Instruments Trading Co., Ltd.) to conduct body composition detection using the bioelectrical impedance method. Operation method: ① on an empty stomach or fasting four hours before the test, strenuous activities were stopped, excrement and urine were emptied, wearing fewer clothes and not carrying heavy items or accessories; ② research subjects stood barefoot on the detector, with both heels and forefoot on the foot electrodes, holding the hand electrodes with both hands, the thumb and the other four fingers were in close contact with the electrodes. The name, age, gender and height were entered, measurement was started after coding, the measurement time was two to three minutes, and body mass index, body fat mass, trunk fat ratio, waist-to-hip ratio (WHR), skeletal muscle and trunk muscle mass were recorded.

The diagnostic criteria for overweight and obesity used height and weight to calculate body mass index (BMI) (weight [Kg] / height [m^2^]). In accordance with the WS/T428-2013 Chinese adult weight judgment standard, average weight is 18.5– 23.99 kg/m^2^, overweight is 24–27.99 kg/m^2^ and obesity is ≥28 kg/m^2^. According to the “Guidelines for the Prevention and Control of Overweight and Obesity in Chinese Adults” published by the Department of Disease Control of Health and Family Planning Commission in 2003, BMI <18.5 kg/m^2^ is considered to be too thin, and 18.5 kg/m^2^ ≤ BMI ≤23.9 kg/m^2^ is considered average, 24.0 ≤ BMI ≤27.9 kg/m^2^ is overweight, BMI ≥28.0 kg/m^2^ is obesity and female waist circumference ≥80 cm is central obesity (9).

### Statistical Methods

SPSS 17.0 statistical analysis software was used for data processing. All measurement data were expressed as mean ± standard deviation (**x** **±** **SD)**. An ANOVA test was used to compare measurement data between groups. A paired *t*-test was used to compare measurement data before and after the intervention. A chi-square test was used to compare count data (and classification data). *P* < 0.05 indicated that the difference was statistically significant.

## Results and Analysis

### General Information

Before the observation, there were no statistical differences in age, educational background and occupational data of groups A, B and C. See Table 1 for *P* > 0.05.

**Table 1:** Comparison of general data.

**Group**	**Number**	**Age($\bar{\text{X}}$ ± s)**	**Degree (Below junior college/above bachelor degree)**	**Profession (Medical/non-medical)**
A	18	49.17 ± 3.22	9/19	4/14
B	28	48.93 ± 3.04	12/16	11/17
C	32	48.13 ± 3.00	18/14	14/18
F(X^2^)		0.839	1.071^X^	2.369^X^
P		0.436	0.585	0.306

X *Random variable*.

### Comparison of Dietary Scores Before and After Different Intervention Methods

Before the intervention, there was no difference in the food intake scores and total scores of the different groups. After the intervention, the intake scores of the three groups of the DASH diet, nuts, soy products, low-fat milk, whole grains, poultry, fish and shrimp white meat, salt and desserts were significantly improved, and the differences were statistically significant (*P* < 0.05). Before and after the intervention, there were no statistical differences in the intake scores of the three groups of fruits and processed meat (*P* > 0.05). Compared with before the intervention, after the intervention, vegetables in group C, processed meat and sweet drinks in groups B and C, the score of red meat intake in group A were increased, the score of red meat intake in group C was significantly decreased, the score of fat and animal viscera intake in groups A and B were significantly increased, and the difference was statistically significant (*P* < 0.05). Compared with group A, the total DASH diet and vegetable intake scores of groups B and C increased significantly, and the low-fat milk intake score of group B increased significantly. The intake scores of nuts, whole grains and desserts in group C were significantly improved, the intake scores of red meat in group C were significantly reduced, the frequency of red meat eating increased, and the difference was statistically significant (*P* < 0.05). After the intervention, the total diet score of group C was higher than A and B, and the difference was statistically significant (*P* < 0.05) (Table 2).

**Table 2:** Comparison of scores of different diet types in different intervention methods.

**Group**	**Number**	**Fruits**		**Vegetables**		**Nuts**		**Soy Products**	
		**Before intervention**	**After intervention**	**Before intervention**	**After intervention**	**Before intervention**	**After intervention**	**Before intervention**	**After intervention**
A	18	3.33 ± 1.029	3.83 ± 0.707	2.44 ± 0.705	2.11 ± 0.471	1.06 ± 1.162	2.56 ± 1.338^*^	1.06 ± 1.11	2.17 ± 1.098^*^
B	28	3.57 ± 0.836	3.68 ± 0.670	2.43 ± 0.742	2.57 ± 0.69^a^	1.18 ± 1.249	2.89 ± 1.166^*^	1.36 ± 0.951	2.96 ± 1.036^*^
C	32	3.66 ± 0.827	3.72 ± 0.683	2.38 ± 0.907	2.781 ± 0.659^a^^*^	1.50 ± 1.368	2.59 ± 1.241^*^	1.53 ± 1.191	3.03 ± 0.897^a^^*^
F/x^2^		1.502	1.844	0.324	11.778	0.836^f^	1.080	2.110^f^	8.458
P		0.472	0.398	0.851	0.003	0.437	0.583	0.348	0.015
**Group**	**Number**	**Low-fat milk**		**Whole grain**		**White meats**		**Salt**	
		**Before intervention**	**After intervention**	**Before intervention**	**After intervention**	**Before intervention**	**After intervention**	**Before intervention**	**After intervention**
A	18	0.94 ± 1.626	2.5 ± 1.618^*^	1.33 ± 1.283	2.11 ± 1.183^*^	0.89 ± 0.676	1.56 ± 0.922^*^	2.22 ± 1.166	2.94 ± 0.802^*^
B	28	0.54 ± 1.071	3.14 ± 1.177^a^^*^	1.39 ± 0.916	2.71 ± 0.976^*^	1.18 ± 1.092	2.04 ± 0.962^*^	1.57 ± 0.879	2.96 ± 0.508^*^
C	32	0.81 ± 1.203	3.00 ± 1.047^*^	1.25 ± 0.950	3.06 ± 0.840^a^^*^	1.25 ± 1.191	2.00 ± 0.95^*^	1.94 ± 1.216	3.31 ± 0.896^*^
F/x^2^		1.019	1.756	0.871	8.757	0.627	4.476	2.035^f^	7.552
P		0.601	0.416	0.647	0.013	0.731	0.107	0.139	0.023
**Group**	**Number**	**Sweet drinks**		**Red meat**		**Processed meat**		**Fatty meat and offal**	
		**Before intervention**	**After intervention**	**Before intervention**	**After intervention**	**Before intervention**	**After intervention**	**Before intervention**	**After intervention**
A	18	3.72 ± 0.575	3.94 ± 0.236	1.94 ± 1.305	2.44 ± 0.784^*^	3.83 ± 0.514	4.0 ± 0.000	3.56 ± 0.511	3.94 ± 0.236^*^
B	28	3.71 ± 0.535	3.93 ± 0.262^*^	2.04 ± 0.999	2.21 ± 0.787	3.71 ± 0.659	4.0 ± 0.000^*^	3.64 ± 0.731	3.93 ± 0.262^*^
C	32	3.59 ± 0.615	3.68 ± 0.336^*^	2.31 ± 1.091	1.84 ± 0.920^a^^*^	3.44 ± 0.982	4.0 ± 0.000^*^	3.59 ± 0.837	3.91 ± 0.39
F/x^2^		1.013	0.848	0.782^f^	5.015	3.628	-	1.434	0.044
P		0.603	0.654	0.461	0.081	0.163	-	0.488	0.978
**Group**	**Number**		**Dessert**				**Total score**		
			**Before intervention**		**After intervention**		**Before intervention**		**After intervention**
A	18		2.78 ± 0.943		3.44 ± 0.705^*^		29.11 ± 5.969		37.56 ± 4.355^*^
B	28		3.18 ± 1.090		3.69 ± 0.723^*^		29.5 ± 5.699		40.71 ± 3.43^a^^*^
C	32		2.81 ± 1.203		3.84 ± 0.369^a^^*^		29.66 ± 7.182		40.97 ± 3.177^a^^*^
F/X^2^			2.954		5.560		0.166^f^		5.951^f^
P			0.228		0.062		0.847		0.004

f *F value; compared with before intervention*,

* *P < 0.05; compared with group A*,

a *P < 0.05*.

### Comparison of Exercise Frequency Before and After Different Intervention Methods

Before the intervention, groups A, B and C exercised irregularly every week, 1–3 times a week, and there was no significant difference in the number of cases where regular exercise was more than three times per week (*P* > 0.05). After the intervention, the number of regular exercises >3 times a week in groups B and C increased significantly, and the difference was statistically significant (*P* < 0.05) (Table 3).

**Table 3:** Comparison of motion frequencies before and after intervention.

**Group**	**Number**	**Before observation**			**After observation**		
		**Irregular exercise**	**1–3 times/per week**	**>3 times /per week**	**Irregular exercise**	**1–3 times/ per week**	**>3 times/per week**
A	18	4 (22.2)	4 (22.2)	10 (55.6)	1 (5.6)	6 (33.3)	11 (61.1)
B	28	6 (21.4)	9 (32.1)	13 (46.4)	1 (3.6)	6 (21.4)	21 (75)^*^
C	32	3 (9.4)	13 (40.6)	16 (50)	0 (0)	4 (12.5)	28 (87.5)^*^
total	78	13 (16.7)	26 (33.3)	37 (47.4)	2 (2.6)	16 (20.5)	60 (76.9)
X^2^		2.082	1.783	0.160	1.941	3.089	5.742
P		0.353	0.410	0.923	0.510	0.213	0.057

*Compared with before treatment*,

* *P < 0.05*.

### Comparison of Changes in the Number of Weekly Aerobic and Resistance Exercises Before and After the Observation of Different Intervention Methods

After the intervention, there was no significant increase in the weekly aerobic exercises in groups A and B, the number of resistance exercises in group A did not change significantly, the amount of aerobic exercise per week in group C was significantly increased, the number of resistance exercises per week in groups B and C increased significantly, and the difference was statistically significant (*P* < 0.05). Comparing the three intervention methods, the weekly aerobic exercises and resistance exercises in group C increased significantly, higher than those in groups A and B, and the difference was statistically significant (*P* < 0.05) (Table 4).

**Table 4:** Comparison of changes in the number of aerobic and resistance exercises per week before and after intervention ($\bar{\text{x}}$ ± SD, Times).

**Group**	**Number**	**Aerobic exercises** **times/week**		**Resistance exercise times/week**	
		**Before intervention**	**After intervention**	**Before intervention**	**After intervention**
A	18	3.61 ± 3.20	3.89 ± 3.18	0.44 ± 1.25	1.28 ± 2.19
B	28	3.07 ± 2.79	3.50 ± 2.46	0.00 ± 0.000	1.21 ± 1.75^*^
C	32	3.72 ± 2.54	5.66 ± 3.03^c^^*^	0.19 ± 0.535	2.72 ± 1.40^c^^*^
*F* value		0.435	4.685	4.536	18.748
*P* value		0.649	0.012	0.104	<0.001

*Compared with before treatment*,

* *P < 0.05; compared with groups A and B*,

c *P < 0.05*.

### Comparison of Waist Circumference, BMI and Body Composition Before and After Different Intervention Methods

After three different intervention methods, body fat and waist circumference were significantly reduced. Skeletal muscle weight was significantly increased. BMI, trunk fat ratios and WHR in groups B and C were significantly reduced. The trunk muscles in group C increased significantly, and the differences were statistically significant (*P* < 0.05). There was no statistical difference in waist circumference, BMI, WHR, body fat mass, trunk fat ratio and muscle mass index between the three intervention methods (*P* > 0.05) (Table 5).

**Table 5:** Waist circumference, body mass index and body composition before and after intervention.

**Group**	**Number**	**Waist circumference (cm)**			**BMI (kg/m** ^**2**^ **)**			**Waist-to-hip ratio**	
		**Before intervention**	**After intervention**		**Before intervention**	**After intervention**		**Before intervention**	**After intervention**
A	18	83.89 ± 9.46	79.08 ± 9.64^*^		25.13 ± 3.18	25.04 ± 3.28		0.91 ± 0.06	0.91 ± 0.06
B	28	83.54 ± 9.49	78.02 ± 8.01^*^		25.28 ± 3.61	24.74 ± 3.48^*^		0.91 ± 0.05	0.90 ± 0.05^*^
C	32	80.83 ± 7.59	75.95 ± 7.72^*^		24.45 ± 3.17	24.09 ± 2.96^*^		0.89 ± 0.39	0.85 ± 0.37^*^
F		1.010	0.935		0.516	0.577		1.988	1.905
P		0.369	0.397		0.599	0.564		0.144	0.156
**Group**	**Number**	**Body fat (kg)**		**Trunk fat ratio (%)**		**Skeletal muscle (kg)**		**Trunk muscles (kg)**	
		**Before intervention**	**After intervention**	**Before intervention**	**After intervention**	**Before intervention**	**After intervention**	**Before intervention**	**After intervention**
A	18	23.35 ± 5.33	22.72 ± 5.55^*^	228.03 ± 56.00	222.75 ± 58.17	22.04 ± 2.52	22.34 ± 2.73^*^	18.62 ± 2.19	18.79 ± 2.24
B	28	23.15 ± 6.13	21.03 ± 6.18^*^	227.88 ± 63.24	210.27 ± 63.35^*^	22.18 ± 3.20	22.41 ±3.02^*^	18.89 ± 2.64	18.94 ± 2.45
C	32	21.00 ± 4.75	19.65 ± 4.48^*^	211.49 ± 54.06	190.91 ± 58.02^*^	21.34 ± 2.38	21.66 ± 2.29^*^	18.03 ± 1.92	18.17 ± 1.85^*^
F		1.618	1.897	0.760	1.182	0.801	0.704	1.135	1.049
P		0.205	0.157	0.471	0.312	0.453	0.498	0.327	0.355

*Compared with before treatment*,

* *P < 0.05*.

## Discussion

Dietary Approaches to Stop Hypertension (DASH), also known as the “DASH diet” model, was proposed in the United States in 1997 as a dietary approach to prevent and control hypertension. It emphasizes the effect of the comprehensive diet model on blood pressure (10, 11). Foreign studies have shown that it has a beneficial effect on blood lipids, diabetes, gestational diabetes and cardiovascular disease (12–14). This study showed that the total score of the DASH diet increased significantly after three different interventions. The frequency of eating nuts, soy products, low-fat milk, whole grains and white meat or poultry, fish and shrimp was significantly increased, and the intake of salt and the frequency of desserts were significantly reduced. After the intervention in group C, the frequency of eating vegetables increased, and the frequency of eating processed meats and sweet drinks decreased significantly. The difference was statistically significant (*P* < 0.05). It shows that different intervention methods can improve the dietary behavior of the population. The dietary behavior of group C improved more obviously, which may be related to the closer communication and contact with the nutritionist in the intensive resistance exercise group twice a week, which played a role in reminding and urging. This study also showed that the frequency of eating red meat in group C was significantly higher than in group A, and the difference was statistically significant (*P* < 0.05). It may be related to the emphasis on high-quality protein intake in the nutrition guidance, and local residents prefer to choose lean pork as a source of high-quality protein. It is necessary to strengthen targeted health education, correct past eating habits, appropriately reduce the frequency of eating red meat and increase the intake of white meat such as chicken, duck, fish and shrimp.

Studies have shown that both diet and intensive exercise intervention can significantly reduce the incidence of metabolic syndrome. Exercise can increase energy consumption in the body, activate the skeletal muscle fatty acid intake system and β oxidation pathways and reduce body fat. Exercise can also increase the uptake and utilization of glucose by skeletal muscle. Exercise can improve lipid metabolism, increase antioxidant enzyme activity, and reduce lipid peroxidation metabolites (15). Weight is affected by many factors, and the fundamental purpose of weight loss is to reduce fat, so the weight change cannot be used to evaluate the effect of weight loss (16). Most people know the causes and hazards of obesity and have the desire to control weight. However, weight control methods are often limited to eating less and exercising, but they cannot persist. Although some obese patients can implement self-weight loss, most patients need help to obtain effective weight loss techniques (17). Health education is the basis of obesity intervention. However, due to the above reasons, pure health education intervention has a certain effect on obese patients, but the actual effect is not obvious. Comprehensive intervention measures of health education + nutrition intervention + exercise intervention should be adopted. Practice has proved that this intervention is a safe, comprehensive intervention method for obese patients (18). In this study, after the intervention, the number of aerobic exercises and resistance exercises in group A did not increase significantly, the number of aerobic exercises per week in group B did not increase significantly, and the number of aerobic exercises and resistance exercises and the total diet score in group C increased significantly, and the difference was statistically significant (*P* < 0.05), indicating that health education + individualized diet guidance + intensive resistance exercise intervention can better improve diet and exercise habits, which is consistent with the results of Yi et al. and Liu and Yuan (16, 19).

Clinical trial studies have confirmed that adipose tissue is the body's largest endocrine organ. It can release dozens of adipocytokines, such as leptin, resistin, adiponectin and tumor necrosis factors. They participate in the occurrence and development of obesity-related diseases by destroying islet 13 cells, inducing hyperinsulinemia and affecting fat metabolism (20). High energy, high fat and high carbohydrate intake may cause body fat accumulation and muscle loss, which is not conducive to the prevention of cardiovascular, cerebrovascular and metabolic diseases. In particular, high energy and high carbohydrate intake should be paid more attention. Low energy intake is not conducive to maintaining muscle mass. High protein intake is not conducive to maintaining minerals. A normal and reasonable ratio of energy intake is most conducive to maintaining the standard body composition (21). Exercise therapy is an important method to prevent and treat obesity, but various exercise methods have different therapeutic effects for patients. Resistance training can improve glucose and fat metabolism. More importantly, resistance training can increase lean body mass and indirectly improve the body fat ratio. Exercise has a positive effect on the prevention and treatment of obesity in postmenopausal women. Medium-intensity aerobic exercise, resistance training or resistance + aerobic exercise are effective ways to lose weight, but the short-term effect is not obvious, and long-term persistence is required (22). In domestic and foreign studies, dietary exercise intervention can effectively control women's body mass, waist circumference, WHR and body composition before and after menopause (23–29). After the intervention of the three different methods in this study, body fat and waist circumference were significantly reduced, skeletal muscle weight was significantly increased, BMI, trunk fat ratio and WHR were significantly decreased in groups B and C. Trunk muscle increased significantly after intervention in group C, and the difference was statistically significant (*P* < 0.05). This indicates that health education + diet guidance + intensive resistance exercise intervention can optimize the body composition of perimenopausal women.

There are shortcomings in this paper. This study used different food intake frequency rules to score the DASH diet. Except for the salt score, it does not score specific food intake and does not record and analyse energy and nutrient intake, which is required to further improve the scoring standards and perfect the design plan. This study records exercise frequency, method and time through WeChat check-ins without introducing information management systems and special equipment to evaluate the compliance and effectiveness of exercise intervention, which may affect the accuracy of the evaluation. The observation period is relatively short, and the number of research cases is small. It is necessary to further increase the sample size, extend the observation time and reduce the research bias.

In summary, although pure health education or health education + dietary intervention can effectively improve diet and exercise habits, reduce waist circumference, body fat, trunk fat and increase skeletal muscle content, the comprehensive intervention of the three methods can give full play to the synergy of each intervention. Health education + diet guidance + intensive resistance exercise interventions can optimize the body composition of perimenopausal women, which is worthy of further promotion and application.

## Data Availability Statement

The original contributions presented in the study are included in the article/supplementary material, further inquiries can be directed to the corresponding author/s.

## Ethics Statement

The studies involving human participants were reviewed and approved by Beijing Pinggu Hospital (2018-District Health Division 001-01). The patients/participants provided their written informed consent to participate in this study. Written informed consent was obtained from the individual(s) for the publication of any potentially identifiable images or data included in this article.

## Author Contributions

SH and ST: designed the study. JingL, WL, and JingyL: analyzed and interpreted the data. XC and ZH: collected and sorted out of data. All authors wrote the manuscript. All authors contributed to the article and approved the submitted version.

## Funding

This work was supported by Scientific Research Project of Beijing Pinggu District Health Commission, project number: pgwjw2018-09.

## Conflict of Interest

The authors declare that the research was conducted in the absence of any commercial or financial relationships that could be construed as a potential conflict of interest.

## Publisher's Note

All claims expressed in this article are solely those of the authors and do not necessarily represent those of their affiliated organizations, or those of the publisher, the editors and the reviewers. Any product that may be evaluated in this article, or claim that may be made by its manufacturer, is not guaranteed or endorsed by the publisher.
